# Erythro-myeloid progenitor origin of Hofbauer cells in the early mouse placenta

**DOI:** 10.1242/dev.200104

**Published:** 2022-04-22

**Authors:** Laina Freyer, Yvan Lallemand, Pascal Dardenne, Alina Sommer, Anne Biton, Elisa Gomez Perdiguero

**Affiliations:** 1 Institut Pasteur, Unit for Macrophages and Endothelial Cells, Developmental and Stem Cell Biology Department, UMR3738 CNRS, 75015 Paris, France; 2 Sorbonne Université, Collège Doctoral, F-75005 Paris, France; 3 Bioinformatics and Biostatistics Hub, Institut Pasteur, 75015 Paris, France

**Keywords:** Developmental hematopoiesis, Erythro-myeloid progenitors, Hofbauer cells, Macrophages, Ontogeny, Placenta

## Abstract

Hofbauer cells (HBCs) are tissue macrophages of the placenta thought to be important for fetoplacental vascular development and innate immune protection. The developmental origins of HBCs remain unresolved and could implicate functional diversity of HBCs in placenta development and disease. In this study, we used flow cytometry and paternally inherited reporters to phenotype placenta macrophages and to identify fetal-derived HBCs and placenta-associated maternal macrophages in the mouse. *In vivo* pulse-labeling traced the ontogeny of HBCs from yolk sac-derived erythro-myeloid progenitors, with a minor contribution from fetal hematopoietic stem cells later on. Single-cell RNA-sequencing revealed transcriptional similarities between placenta macrophages and erythro-myeloid progenitor-derived fetal liver macrophages and microglia. As with other fetal tissue macrophages, HBCs were dependent on the transcription factor Pu.1, the loss-of-function of which in embryos disrupted fetoplacental labyrinth morphology, supporting a role for HBC in labyrinth angiogenesis and/or remodeling. HBC were also sensitive to *Pu.1* (*Spi1*) haploinsufficiency, which caused an initial deficiency in the numbers of macrophages in the early mouse placenta. These results provide groundwork for future investigation into the relationship between HBC ontogeny and function in placenta pathophysiology.

## Abstract (precis)

**Summary:** Feto-placental macrophages called Hofbauer cells are functionally distinct from maternal placenta macrophages, originate from yolk-sac erythro-myeloid progenitors and are controlled by Pu.1 in a dose-dependent manner.

## INTRODUCTION

The hemochorial placenta is a uniquely chimeric organ formed from maternal and fetal tissue in which maternal blood comes in direct contact with the chorion, a feature common to rodent and primate placentation. An abundance of circulating maternal immune cells are found in the placenta, although placenta macrophages called Hofbauer cells (HBCs) are the only fetal immune cells located within the chorionic stroma (Takahashi et al., 1991; Hoo et al., 2020). HBC express high levels of growth factors that support angiogenesis, branching morphogenesis and tissue remodeling (Demir et al., 2004; Anteby et al., 2005; Plaks et al., 2013), implying a role for HBC in shaping the fetoplacental vascular bed that is essential for blood flow, nutrient exchange and fetal growth (Burton et al., 2016; Woods et al., 2018). HBCs may also provide *in utero* innate immune protection against infections, although HBC hyperplasia is associated with viral infections, inflammatory conditions and pregnancy complications, such as villitis, chorioamnionitis, pre-eclampsia and pre-term birth (Thomas et al., 2021; Satosar et al., 2004; Rosenberg et al., 2017; Reyes and Golos, 2018; Zulu et al., 2019; Mezouar et al., 2020).

Evidence supports the notion that HBCs share ontogeny with yolk-sac macrophages, exemplified by their structural, functional and transcriptomic similarities (Takahashi et al., 1991; Thomas et al., 2021). Yolk-sac macrophages originate from erythro-myeloid progenitors (EMPs) that are generated from the yolk-sac endothelium (Palis et al., 1999; Lux et al., 2008; Frame et al., 2016; Kasaai et al., 2017). EMP-derived macrophage precursors migrate via the peripheral circulation to seed embryonic tissues, a process that coincides with the first appearance of infiltrating HBCs in the mouse chorionic stroma (Takahashi et al., 1991; Stremmel et al., 2018). However, it has also been suggested that circulating fetal monocytes originating from fetal hematopoietic stem cells (HSCs) may infiltrate the chorionic stroma to give rise to HBCs at later stages of development (Mezouar et al., 2020).

The ontogeny of tissue macrophages is important because developmental origins impart unique functional characteristics with respect to inflammation and tissue remodeling (Loyher et al., 2018; Zhu et al., 2017; Honold and Nahrendorf, 2018). Placenta macrophage diversity changes with gestational age and whether this is due to developmentally distinct origins of HBCs is yet to be determined. Therefore, there is a newfound appreciation for the ontogeny of HBCs as an emerging field that will shed light on the roles of HBCs in placenta development and disease (Mezouar et al., 2020; Zulu et al., 2019).

## RESULTS AND DISCUSSION

### Isolation of fetal Hofbauer cells and their precursors from the mouse placenta

**Fig. 1.**
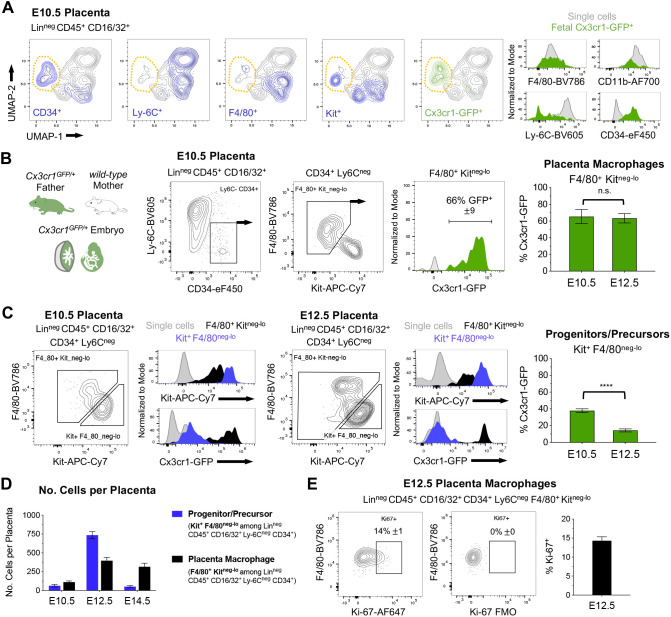
**Immunophenotyping of mouse placenta macrophages and precursors.** (A) UMAP dimensionality reduction and histograms of flow cytometry data from the E10.5 placenta based on 14 parameters (see Materials and Methods) applied to Lin^neg^ (Ter119^neg^ CD19^neg^ CD8^neg^ CD4^neg^ CD3e^neg^ NK1.1^neg^ Ly6G^neg^) CD45^+^ CD16/32^+^ cells. Expression of individual markers is highlighted in blue. A cluster of placenta macrophages (orange outline) expresses the paternally inherited *Cx3cr1^GFP^* reporter. (B) Isolation of placenta macrophages from *Cx3cr1^GFP/+^* embryos (*Cx3cr1^GFP/+^* father crossed to wild-type mother, see Fig. S1 for gating strategy). Data are mean±s.e.m of *n=*9 embryos from three experiments (E10.5) and *n=*5 embryos from two experiments (E12.5). (C) Isolation of progenitors/precursors (see Fig. S1 for gating strategy). Histograms show low expression of *Cx3cr1^GFP^* among progenitors/precursors at E10.5 and E12.5 compared with placenta macrophages (F4/80^+^ Kit^neg-lo^) or single cells (see Fig. S1A for gating strategy). Data are mean±s.e.m of *n=*9 embryos from three experiments (E10.5) and *n=*5 embryos from two experiments (E12.5). (D) Number of placenta macrophages and progenitors/precursors per placenta at E10.5, E12.5 and E14.5. Data are mean±s.e.m of *n=*9 embryos from three experiments (E10.5), *n=*13 embryos from four experiments (E12.5) and *n=*10 embryos from three experiments (E14.5). (E) Flow cytometry analysis of the proliferation marker Ki-67 among placenta macrophages at E12.5. FMO, fluorescence minus one. Data are mean±s.e.m of *n=*12 embryos from two experiments. ****P<0.0001; n.s., not significant (Student's unpaired *t-*test with Welch's correction).

In order to isolate HBC from the mouse placenta, we took advantage of the *Cx3cr1^GFP^* reporter strain that expresses GFP in fetal macrophages and circulating fetal monocytes (Jung et al., 2000; Hoeffel et al., 2015). By flow cytometry, we analyzed the E10.5 and E12.5 placenta and found that fetal-derived Cx3cr1-GFP^+^ cells were largely underrepresented among total mononuclear phagocytic cells (monocytes and macrophages) due to the abundance of maternal myeloid cells (Fig. S1A,B). To further isolate placenta macrophages, we developed a gating strategy based on UMAP dimensionality reduction of flow cytometry data (Fig. 1A). This allowed us to select a population of immunophenotypic placenta macrophages (Lin^neg^ CD45^+^ CD16/32^+^ Ly6C^neg^ CD34^+^ F4/80^+^ Kit^neg-lo^) that were enriched for Cx3cr1-GFP^+^ fetal-derived HBCs (Fig. 1B, Fig. S1B). *Cx3cr1-GFP* was also expressed at lower levels among a population of progenitors/precursors (Lin^neg^ CD45^+^ CD16/32^+^ Ly6C^neg^ CD34^+^ F4/80^neg-lo^ Kit^+^), suggesting that this compartment was populated by macrophage precursors that have been previously described (Takahashi et al., 1991; Bertrand et al., 2005) (Fig. 1C, Fig. S1C). The numbers of progenitors/precursors was highest at E12.5, corresponding to the peak in hematopoietic stem and progenitor cells observed at this stage (Gekas et al., 2005; Ottersbach and Dzierzak, 2005) (Fig. 1D). Yet only a fraction of these were Cx3cr1-GFP^lo^, suggesting that macrophage precursors were primarily present at earlier stages (Fig. 1C). The number of placenta macrophages increased with time (Fig. 1D) and a high proportion expressed the proliferation marker Ki-67 at E12.5 (Fig. 1E, Fig. S1D), indicating that they may proliferate *in situ* as described for other tissue macrophages.

### Maternal contribution to immunophenotypic placenta macrophages

**Fig. 2.**
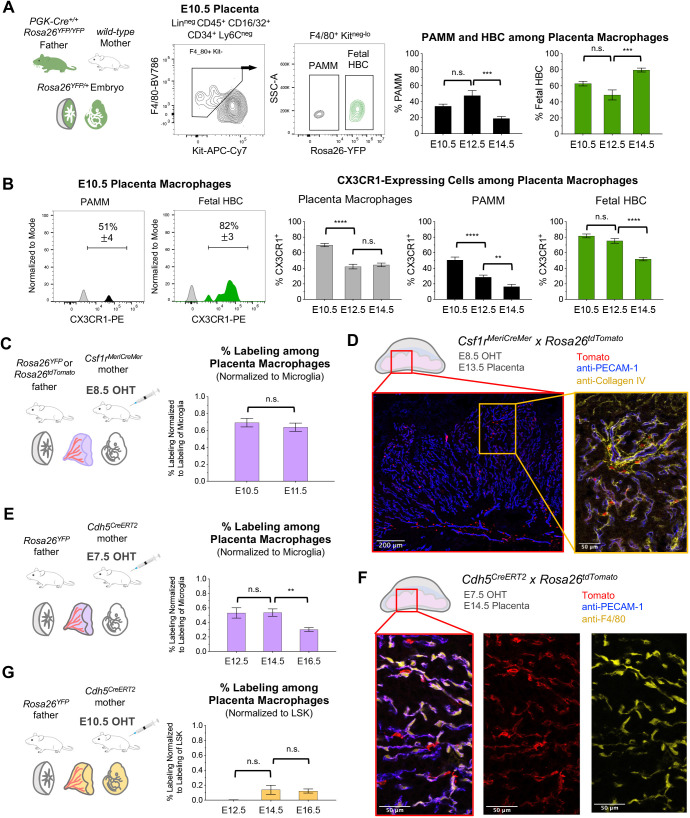
**Mixed ontogeny of mouse placenta macrophages.** (A) Labeling of all fetal cells using a paternally inherited *Rosa26^YFP^* allele (recombined in a previous generation by *PGK-Cre*). Fetal HBCs were isolated from PAMMs based on expression of YFP. Quantification of maternal and fetal cells among the placenta macrophage gate at E10.5, E12.5 and E14.5. Data are mean±s.e.m of *n=*22 from four experiments (E10.5), *n=*12 from two experiments (E12.5) and *n=*12 from two experiments (E14.5). (B) Histograms from the E10.5 placenta showing expression of CX3CR1, as detected by antibody. Proportion of placenta macrophages expressing CX3CR1 further divided into PAMM (YFP^neg^) and fetal HBC (YFP^+^) subsets at E10.5, E12.5 and E14.5. Data are mean±s.e.m of *n=*22 from four experiments (E10.5), *n=*12 from two experiments (E12.5) and *n=*12 from two experiments (E14.5). (C) Pulse labeling of E10.5 and E11.5 placenta macrophages from *Csf1r^MeriCreMer^ Rosa26^YFP^* or *Rosa26^tdTomato^* embryos with E8.5 OHT. Data are mean±s.e.m of *n=*6 embryos from two experiments (E10.5) and *n=*17 embryos from three experiments (E11.5). (D) Immunofluorescence on E13.5 placenta from *Csf1r^MeriCreMer^ Rosa26^tdTomato^* embryos with E8.5 OHT using antibodies to PECAM1 and collagen IV. (E) Pulse-labeling of E12.5, E14.5 and E16.5 placenta macrophages from *Cdh5^CreERT2^ Rosa26^YFP^* embryos with E7.5 OHT. Data are mean±s.e.m of *n=*6 from two experiments (E12.5), *n=*9 from two experiments (E14.5) and *n=*11 from two experiments (E16.5). (F) Immunofluorescence on E14.5 placenta from *Cdh5^CreERT2^ Rosa26^tdTomato^* embryos with E7.5 OHT using antibodies to PECAM-1 and F4/80. (G) Pulse-labeling of E12.5, E14.5 and E16.5 placenta macrophages from *Cdh5^CreERT2^ Rosa26^YFP^* embryos with E10.5 OHT. Data are mean±s.e.m of *n=*8 from two experiments (E12.5), *n*=9 from two experiments (E14.5) and *n=*8 from two experiments (E16.5). **P<0.01; ***P<0.001; ****P<0.0001; n.s., not significant (Student's unpaired *t-*test with Welch's correction).

A proportion of placenta macrophages did not express the *Cx3cr1^GFP^* reporter (Fig. 1B), possibly due to heterogeneous expression of *Cx3cr1^GFP^* among HBC or mixed origins of placenta macrophages. For example, placenta-associated maternal macrophages (PAMMs) are maternal macrophages that adhere to the lining of the chorion and are captured when isolating HBC from the human chorionic stroma (Thomas et al., 2021). To address these possibilities, we used an antibody to CX3CR1, together with a ubiquitously expressed paternally inherited fluorescent reporter that definitively marked all fetal cells as YFP^+^ (Fig. 2A,B). This revealed that the placenta macrophage compartment was a mixed population of maternal (YFP^neg^ PAMMs) and fetal (YFP^+^ HBCs) macrophages, the relative contributions of which were dynamic over time (Fig. 2A). This was confirmed using an additional fluorescent reporter strain to label all maternal cells as YFP^+^, while marking all fetal cells as mTomato^+^ (Fig. S2A). Both PAMMs and HBCs exhibited heterogeneous expression of CX3CR1 (Fig. 2B), thus accounting for the incomplete labeling of placenta macrophages by the *Cx3cr1^GFP^* reporter, even when both the mother and fetus expressed the *Cx3cr1^GFP^* allele (Fig. S2B,C). Future investigation into HBC- or PAMM-specific markers could provide alternative means to separate these populations.

### Shared ontogeny between Hofbauer cells and yolk-sac erythro-myeloid progenitors

To investigate the contribution of EMP and HSC hematopoiesis to HBCs, we performed *in vivo* pulse-labeling. Previous work has demonstrated that drug-inducible mouse strains, such as *Csf1r^MeriCreMer^* and *Cdh5^CreERT2^*, can be used to trace the ontogeny of fetal blood and immune cells from functionally distinct waves of EMPs or HSCs (Ginhoux et al., 2010; Schulz et al., 2012; Gomez Perdiguero et al., 2015; Hoeffel et al., 2015; Gentek et al., 2018; Soares-Da-Silva et al., 2021; Freyer et al., 2020 preprint). We crossed *Csf1r^MeriCreMer^* mice to *Rosa26^YFP^* or *Rosa26^Tomato^* reporter strains and injected 4-hydroxytamoxifen (OHT) at E8.5 to label yolk sac EMP and their progeny (Fig. 2C,D). As drug-inducible Cre-mediated excision is not 100% penetrant and as different reporter alleles have unique recombination efficiencies, we used the labeling of microglia (efficiently targeted by EMP pulse-labeling) as a proxy for the overall efficiency of pulse-labeling experiments (Fig. S2D). Placenta macrophages were efficiently labeled at E10.5 and E11.5, thus supporting an EMP origin of HBCs (Fig. 2C). These results were confirmed at E12.5, E14.5 and E16.5 using *Cdh5^CreERT2^* mice crossed to *Rosa26^YFP^*, which also labels the EMP wave of hematopoiesis when OHT is injected at E7.5 (Fig. 2E,F, Fig. S2E). This was in line with recent findings demonstrating the EMP origin of IBA1^+^ placenta macrophages in the E17.5 mouse placenta (Ceasrine et al., 2021 preprint).

Pulse labeling of placenta macrophages by *Cdh5^CreERT2^* with E7.5 OHT significantly decreased at E16.5 (Fig. 2E), coinciding with tissue infiltration by circulating fetal monocytes (Hoeffel et al., 2015) that can originate from either EMPs or HSCs (Freyer et al., 2020 preprint). We used *Cdh5^CreERT2^ Rosa26^YFP^* mice with E10.5 OHT to trace the HSC wave of hematopoiesis. In this case, labeling efficiency was normalized to LSK (Lin^neg^ Sca-1^+^ Kit^+^), which includes the fetal HSC compartment targeted by this approach (Fig. S2F). HSC-derived cells contributed minimally to placenta macrophages from E14.5 to E16.5 (Fig. 2G). The remaining placenta macrophages that were not efficiently targeted by either of these approaches may be accounted for by the presence of PAMMs among placenta macrophages and will require future investigation to definitively label all fetal cells in combination with pulse labeling.

### Transcriptional profile of mouse placenta macrophages

**Fig. 3.**
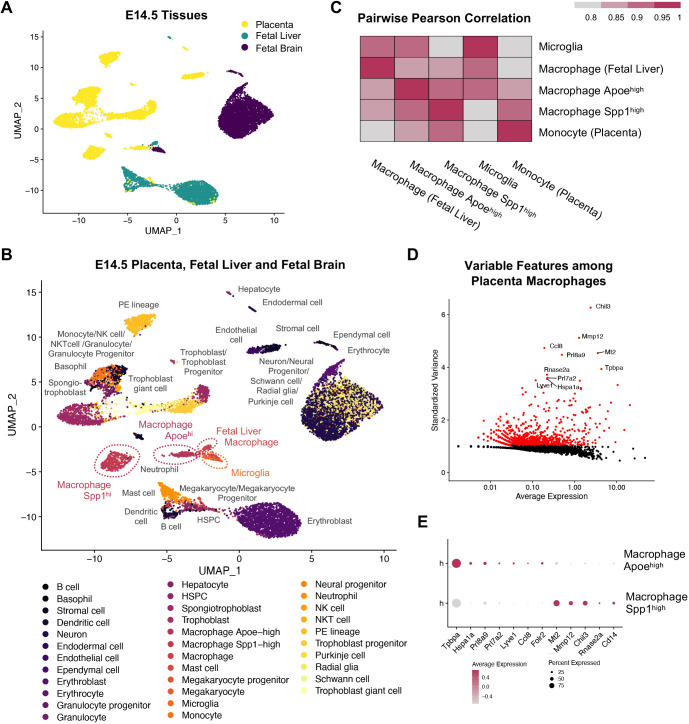
**Molecular profiles of E14.5 mouse placenta macrophages.** (A) Integration of single-cell RNA-sequencing data from E14.5 mouse placenta, fetal liver and fetal brain (data provided by the Mouse Cell Atlas). (B) Clustering of Apoe^high^ placenta macrophages with fetal liver macrophages and microglia. (C) Pairwise Pearson correlation coefficient between average cluster expression profiles. (D) Top 10 variable features when comparing Apoe^high^ and Spp1^high^ placenta macrophages. (E) Dot plot representing average expression and percentage expression of the top 10 variable features among Apoe^high^ and Spp1^high^ placenta macrophages.

To gain more insight into the molecular profiles of mouse placenta macrophages, we analyzed single-cell RNA-sequencing data from the Mouse Cell Atlas, including two distinct populations of macrophages (Apoe^high^ and Spp1^high^) from the E14.5 placenta (Han et al., 2018) (Fig. S3A). We compared Apoe^high^ and Spp1^high^ placenta macrophages to other fetal tissue macrophages and found that Apoe^high^ placenta macrophages clustered with fetal liver macrophages and microglia and were highly correlated to microglia, whereas Spp1^high^ placenta macrophages correlated with monocytes (Fig. 3A-C). Apoe^high^ macrophages, fetal liver macrophages and microglia expressed high levels of *Selenop* and *Apoe* along with components of the complement system (*C1qc* and *C1qa*) and had a higher proportion of cells assigned to the G2M phase of the cell cycle compared with Spp1^high^ macrophages or monocytes (Fig. S3B,C). When comparing Apoe^high^ versus Spp1^high^ macrophages, the most variable genes were *Tpbpa*, *Hspa1a*, *Prl8a9*, *Prl7a2*, *Lyve1* and *Ccl8* (expressed among Apoe^high^ macrophages), and *Mt2*, *Mmp12*, *Chil3* and *Rnase2a* (expressed among Spp1^high^ macrophages), in addition to differential expression of *Folr2* and *Cd14*, as reported in the human placenta (Thomas et al., 2021) (Fig. 3D,E).

### Requirement for *Pu.1* among placenta macrophages

**Fig. 4.**
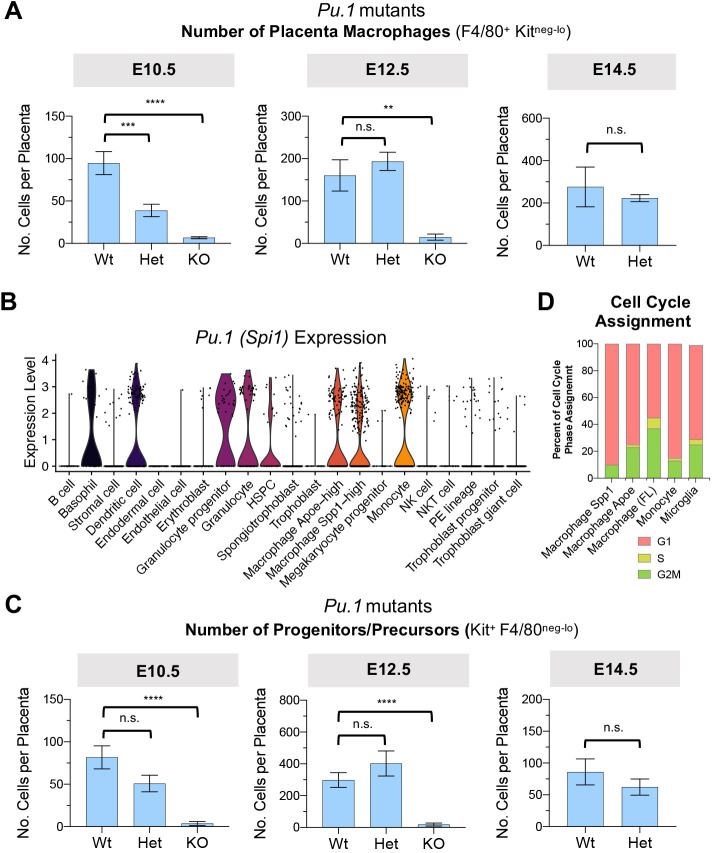
***Pu.1* dose-dependent dynamics of mouse placenta macrophages.** (A) Quantification of placenta macrophages from E10.5, E12.5 and E14.5 placentas of *Pu.1* mutant embryos. (B) Expression of *Pu.1* (*Spi1*) among cell types of the E14.5 placenta (see Fig. S3A). (C) Quantification of progenitors/precursors from E10.5, E12.5 and E14.5 placentas of *Pu.1* mutant embryos. (D) Cell cycle phase classification based on gene expression using cyclone. Proportions of cells from mononuclear phagocytic clusters assigned to phases of cell cycle. Data are mean±s.e.m of *n=*25 (*Pu.1^+/+^* Wt) and *n*=25 (*Pu.1^+/−^* Het) embryos from six experiments (E10.5); *n=*7 (*Pu.1^−/−^* KO) from three experiments (E10.5); *n=*16 (*Pu.1^+/+^* Wt) and *n*=13 (*Pu.1^+/−^* Het) embryos from three experiments (E12.5); *n=*3 (*Pu.1^−/−^* KO) embryos from two experiments (E12.5); and *n=*4 (*Pu.1^+/+^* Wt) and *n*=12 (*Pu.1^+/−^* Het) embryos from two experiments. **P<0.01, ***P<0.001, ****P<0.0001, n.s., not significant (Student's unpaired *t-*test with Welch's correction).

To explore functional requirements for HBC, we used mice that were mutant for the Pu.1 transcription factor that is essential for development of yolk sac tissue macrophages (McKercher et al., 1996; Schulz et al., 2012). *Pu.1* (*Spi1*) null embryos fail to develop macrophages, although they appear grossly normal *in utero* (McKercher et al., 1996), providing an opportunity to assess the placenta without compounding embryonic defects. E12.5 placentas from *Pu.1^−/−^* null embryos had poorly vascularized labyrinths lacking in macrophages (Fig. S4A), supporting the notion that HBCs are required for vasculogenesis and/or tissue remodeling. Macrophages were still observed in the maternal blood sinus of placentas from *Pu.1^−/−^* embryos and further work is needed to characterize the role of PAMMs in the absence of HBCs. Haploinsufficiency of fetal *Pu.1* also resulted in a significant loss of placenta macrophages at E10.5 (Fig. 4A), similar to what was observed for yolk-sac macrophages (Schulz et al., 2012). There were no significant differences in the numbers of placenta macrophages at E12.5 and E14.5 among *Pu.1* wild-type and heterozygous mutants, indicating that compensatory mechanisms worked to restore the total number of placenta macrophages (Fig. 4A). Hematopoietic stem and progenitor cells (HSPC) also express *Pu.1* (Fig. 4B) and the numbers of progenitors/precursors were moderately affected by heterozygous and homozygous loss of fetal *Pu.1* expression at E10.5 (Fig. 4C). Therefore, it is not possible to distinguish the role of Pu.1 in HSPC versus placenta macrophages using this approach, although the proliferative capacity of HBC (Figs 1E and 4D) suggests a mechanism to overcome initial deficiencies in HBCs. Further investigations into the mechanisms that control HBC proliferation will be important for studies of HBC hyperplasia associated with infection and inflammatory conditions.

### Conclusions

Mouse models are invaluable to study the ontogeny and function of HBCs as they share core features with human placentation (Hemberger et al., 2020), while making it possible to label cells with spatiotemporal precision. In this report, we used fluorescent reporter strains and flow cytometry strategies to separate HBCs from PAMMs. Using two independent *in vivo* pulse-labeling models, we concluded that yolk sac EMPs contributed significantly to HBCs in the mouse placenta, with a smaller contribution from HSC-derived cells at later developmental timepoints. A subset of molecularly distinct placenta macrophages shared similarities with other fetal tissue macrophages, such as microglia and fetal liver macrophages. Development of HBCs required expression of the Ets family transcription factor Pu.1 and loss of HBC in the placenta of *Pu.1*-null mutants resulted in labyrinth abnormalities. The establishment of the HBC population in the early mouse placenta also required *Pu.1* in a dose-dependent manner with a marked reduction of HBCs in the placenta of *Pu.1* heterozygous mutants that recovered with time. In the future, *Pu.1* mutant analysis will be combined with the ubiquitous paternal reporter to further characterize the requirement for HBCs in the chorionic stromal environment under normal and inflammatory states, to determine how loss of HBCs impacts the presence of PAMMs and to investigate mechanisms by which HBCs regulate vascular development.

Further work is needed to determine the contribution of infiltrating monocytes to HBCs. As circulating fetal monocytes can originate from multiple waves of hematopoiesis (Hoeffel et al., 2015; Freyer et al., 2020 preprint), alternative models will be needed to trace circulating fetal monocytes regardless of their EMP or HSC origins. These findings will be important for comparison with human HBCs that are often collected from full-term pregnancies but may be functionally heterogeneous compared with early HBCs. It will be of interest to study whether HBC ontogeny is a determining factor that influences how placenta macrophages respond to infection or inflammation, and how this shapes the placenta microenvironment over time.

## MATERIALS AND METHODS

### Mice

Experimental procedures, housing and husbandry of mice were conducted in compliance with the regulatory guidelines of the Institut Pasteur Committee for Ethics and Animal Experimentation (CETEA, dap160091). Mouse strains have been previously described: *Cx3cr1^GFP^* (Jung et al., 2000), *PGK-Cre* (Lallemand et al., 1998), *Rosa26^YFP^* (Srinivas et al., 2001), *Rosa26^tdTomato^* (Madisen et al., 2010), *Rosa26^mTmG^* (Muzumdar et al., 2007), *Csf1r^MeriCreMer^* (Qian et al., 2011), *Cdh5^CreERT2^* (Sörensen et al., 2009) and *Pu.1* (McKercher et al., 1996). For staged embryos, timed matings were performed and the date of vaginal plug was considered to be embryonic day (E)0.5.

### Flow cytometry

Tissues were dissected in ice-cold PBS and cells were dissociated in digestion buffer (PBS with 1 mg/ml collagenase D (Sigma-Aldrich 11088882001), 100 U/ml DNaseI (DN25-100 mg) and 3% fetal bovine serum) for 30 min at 37°C then mashed through a 100 µm strainer using a syringe piston. Placentas were minced with scissors prior to digestion. Cells were collected in FACS buffer (0.5% BSA and 2 mM EDTA in PBS) and pelleted by centrifugation at 320 ***g*** for 7 min. Blocking was performed with 5% FBS and 1:20 mouse IgG (Interchim, 015-000-003) in FACS buffer followed by 30 min of antibody staining. A list of fluorescently conjugated antibodies used for flow cytometry along with details about the clones, concentrations, manufacturers and reference numbers is provided in the Table S1. Cells were washed and incubated with fluorescently conjugated streptavidin for 20 min. Stained cells were passed on the BD Symphony A5 cytometer with Diva software or a Beckman Cytoflex LX with CytExpert software. The formula (number of cells acquired)×(volume of resuspended cells after staining and washing/volume of cells acquired)×(volume of cell suspension in blocking buffer prior to staining/volume of cells plated for staining) was used to quantify the numbers of cells per tissue. Results were analyzed and plots generated using FlowJo 10.8.1 software. Gating strategies are provided in the supplementary Materials and Methods. The UMAP v3.1 plugin of FlowJo 10.8.1 software was used with default parameters; Euclidean distance function with nearest neighbors=15, minimum distance=0.5 and number of components=2 (McInnes et al., 2018). Compensated parameters for CD71-AF647, CD11b-AF700, Kit-APC-Cy7, CD34-eF450, Sca-1-BV510, Ly-6C-BV605, F4/80-BV786, CD41-BUV395, CD45-BUV661, CD16/32-BUV737, Flt3-PE, CD115-PE-CF594 and Itgb7-PE-Cy7 were selected from Lin^neg^ (Ter119^neg^ CD19^neg^ CD8^neg^ CD4^neg^ CD3e^neg^ NK1.1^neg^ Ly6G^neg^) CD45^+^ CD16/32^+^ cells.

### Intracellular staining

To assess Ki-67 expression, cells were collected for flow cytometry and stained for cell surface proteins as described above. Cells were then fixed in 4% paraformaldehyde for 15 min on ice, washed, permeabilized and blocked in 0.1% saponin with 2% normal goat serum for 15 min on ice, incubated with anti-Ki67 (1:50) in 0.1% saponin for 1 h on ice, washed in 0.1% saponin then resuspended in FACS buffer.

### Immunofluorescence

Placentas were fixed for 4 h at 4°C in 4% paraformaldehyde in PBS then washed three times in PBS. Whole tissues were cryoprotected in 30% sucrose, embedded in OCT (TissueTek) then cryosectioned at 12 μm. Cryosections were air-dried for 10 min then permeabilized with 0.5% Triton X-100 in PBS for 5 min. Sections were blocked with 10% normal goat serum for 2 h at room temperature followed by consecutive overnight incubations at 4°C with primary and secondary antibodies diluted in blocking buffer (Table S1). Sections were washed 3×10 min in 0.1% Triton X-100 in PBS after each antibody incubation and coverslipped with Prolong Gold Antifade.

### Single-cell RNA-sequencing analysis

Raw data files for the RNA-sequencing can be accessed at GEO (GSE108097). Digital Expression Matrixes are available at https://figshare.com/s/865e694ad06d5857db4b, with an R package for scMCA analysis on GitHub (https://github.com/ggjlab/scMCA) as reported in Han et al. (2018). Data from the Mouse Cell Atlas (Han et al., 2018) were provided at https://satijalab.org/seurat/v3.0/mca.html. Analysis was performed using Seurat 4.0.2. Cells from tissues of interest (placenta, fetal liver and fetal brain) were selected using the subset function. Data were normalized, scaled, clustered and UMAP dimensionality reduction performed. Some of the cluster annotations were renamed in order to simplify the labeling of the UMAP plot in Fig. 3B. Mononuclear phagocytic cells were further subdivided to generate a heatmap of the pairwise Pearson correlation coefficient between the average cluster expression profiles and to compare variable features among placenta macrophages. Cell cycle phase classification was performed using cyclone (Scialdone et al., 2015).

### 4-OHT injections

OHT (4-hydroxytamoxifen; Sigma-Aldrich, H7904-25MG) was dissolved to 50 mg/ml in equal volumes of ethanol and Kolliphor (Sigma-Aldrich C5135-500G) with sonication. 25 mg of progesterone (P3972) was resuspended in 250 µl of ethanol and 2250 µl of sunflower oil (Sigma-Aldrich S5007-250ML). Females from timed matings were co-injected with OHT (75 µg per gram of body weight for *Csf1r^MeriCreMer^* or 50 µg per gram of body weight for *Cdh5^CreERT2^*) and progesterone (37.5 µg per gram of body weight for *Csf1r^MeriCreMer/+^* or 25 µg per gram of body weight for *Cdh5^CreERT2/+^*) prepared in a solution of 0.9% NaCl. Injections were performed at 13 h according to the weight of the females on day 7 of pregnancy.

## Supplementary Material

Supplementary information
