# Supplementary material for: Erythro-myeloid progenitor origin of Hofbauer cells in the early mouse placenta
Source: Development. 2022 Apr 22;149(8):dev200104. doi: 10.1242/dev.200104 (PMC9124577; doi:10.1242/dev.200104)
Supplement: Supplementary information [file develop-149-200104-s1.pdf]

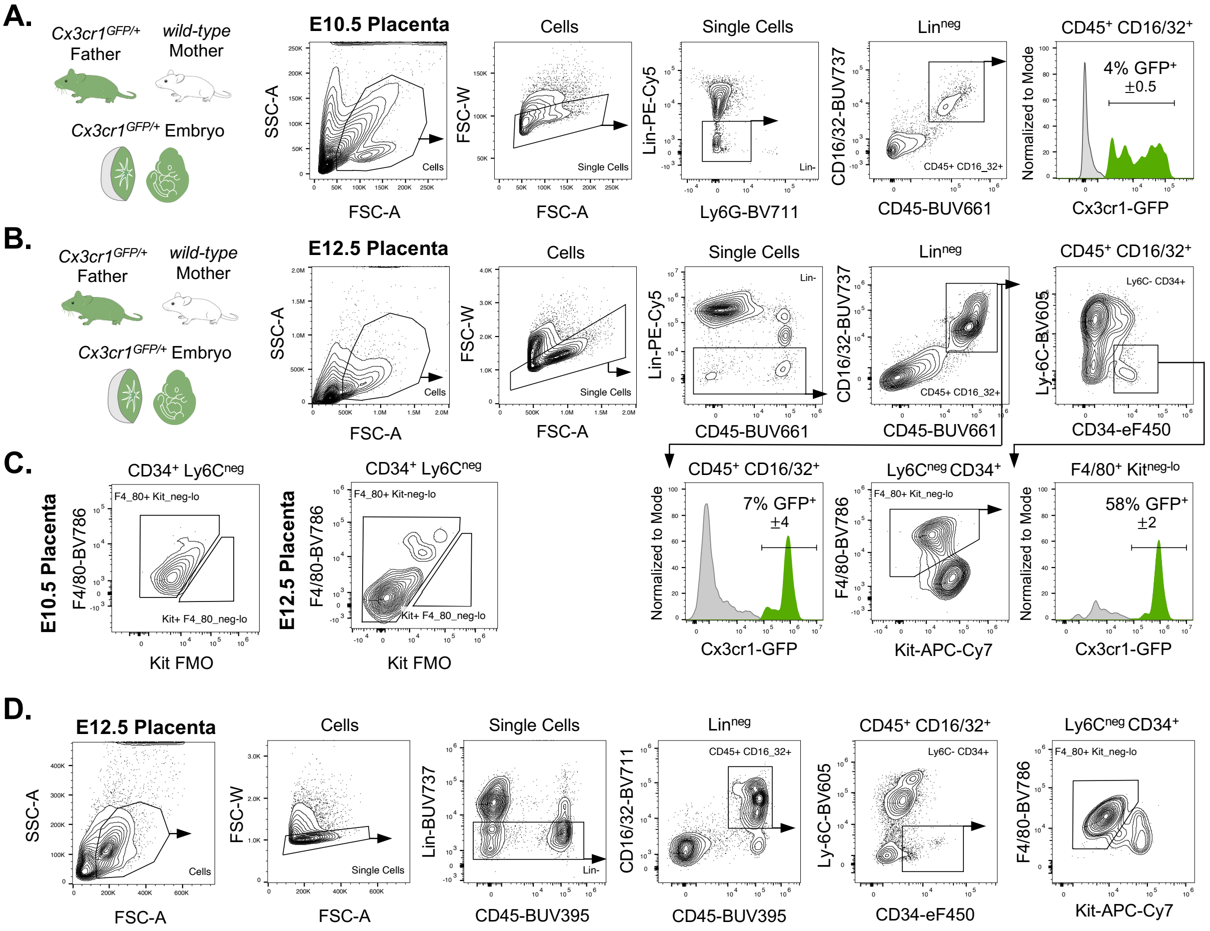

Fig. S1. Isolation of fetal placenta macrophages and precursors.

(**A**) Gating strategy for placenta macrophages from the E10.5 placenta of *Cx3cr1*$^{GFP/+}$ embryos (*Cx3cr1*$^{GFP/+}$ father crossed to *wild-type* mother, related to Fig. 1A-C). In order to isolate monocytes and macrophages, lymphoid cells and granulocytes were excluded. Only 4% of total Lin^{neg} (Ter119^{neg} CD19^{neg} CD8^{neg} CD4^{neg} CD3e^{neg} NK1.1^{neg} Ly6G^{neg}) CD45^{+} CD16/32^{+} mononuclear phagocytes express the *Cx3cr1*^{GFP} reporter at E10.5. (**B**) Gating strategy for isolation of placenta macrophages from E12.5 *Cx3cr1*$^{GFP/+}$ embryos (*Cx3cr1*$^{GFP/+}$ father crossed to *wild-type* mother, related to Fig. 1B-C). (**C**) Fluorescence Minus One (FMO) controls for Kit-APC-Cy7 staining of placenta macrophage progenitors and/or precursors (Lin^{neg} CD45^{+} CD16/32^{+} Ly-6C^{neg} CD34^{+} Kit^{+} F4/80$^{neg\text{-}lo}$) at E10.5 and E12.5 (related to Fig. 1C-D). (**D**) Gating strategy for flow cytometry analysis of fixed cells for the quantification of Ki-67 expression among E12.5 placenta macrophages (related to Fig. 1E).

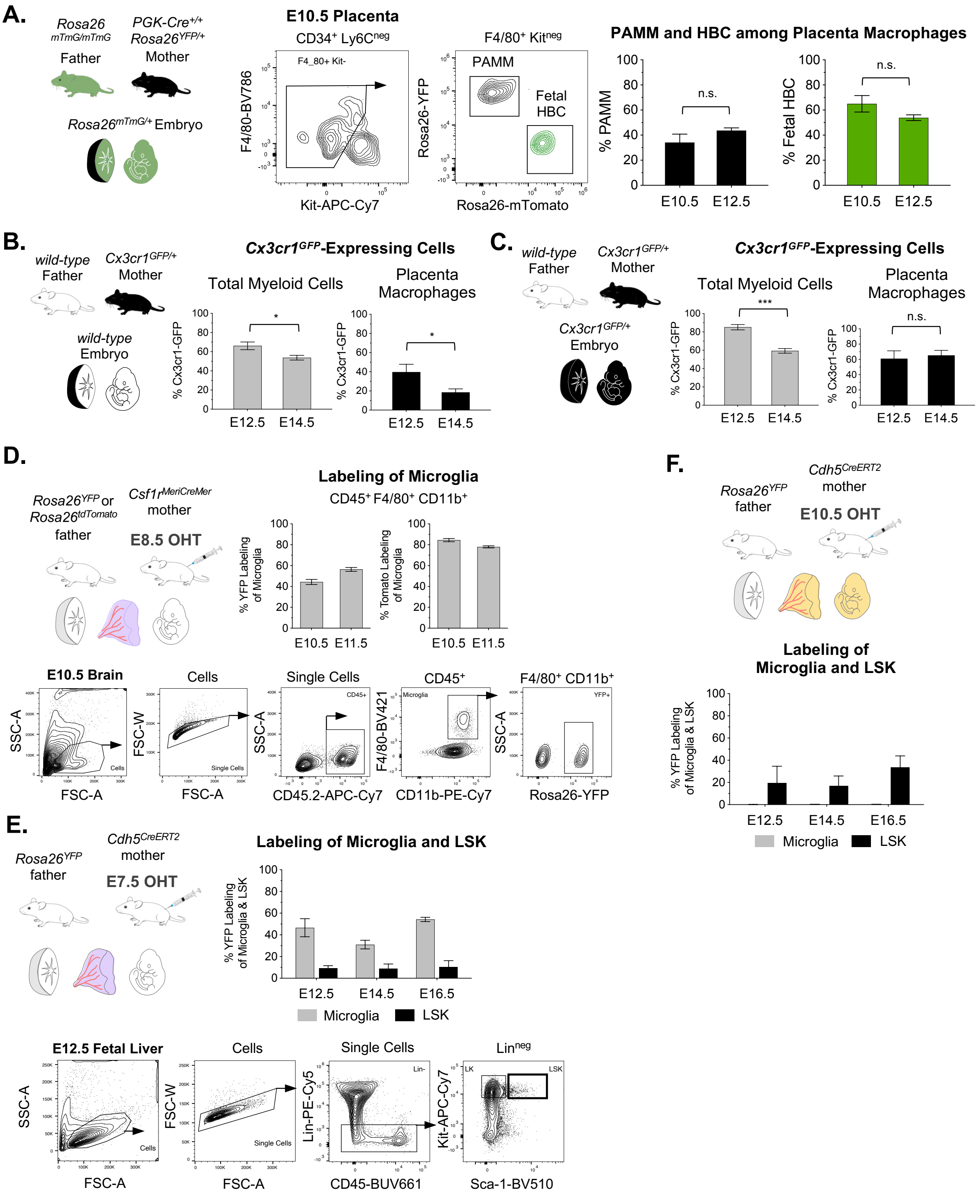

Fig. S2. Isolation of maternal placenta macrophages and pulse labeling of EMP- and HSC-derived macrophages. (**A**) Mating strategy to label all maternal cells using a maternally expressed allele of the *Rosa26*^{YFP} locus (recombined in a previous generation by the ubiquitously expressed *PGK-Cre* transgene and not inherited by the embryo) together with labeling of all fetal cells using a paternally inherited allele of the *Rosa26*^{mTmG} locus (ubiquitously expresses mTomato without recombination). Separation of PAMM (YFP^{+}

Development • Supplementary information

mTomato^{neg}) from fetal HBC (mTomato^{+} YFP^{neg}). Representative gating strategy from E10.5 placenta. Quantification of the proportions of PAMM and fetal HBC among E10.5 and E12.5 placenta macrophages (related to Fig. 2A). Data are represented as mean ± s.e.m of n = 8 embryos from one experiment (E10.5) and n = 8 embryos from one experiment (E12.5). (**B**) Expression of the *Cx3cr1*^{GFP} reporter in maternal placenta macrophages when the embryo has not inherited the *Cx3cr1*^{GFP} allele. Data are represented as mean ± s.e.m of n = 7 embryos from two experiments (E12.5) and n = 6 embryos from three experiments (E14.5). (**C**) Expression of the *Cx3cr1*^{GFP} reporter in placenta macrophages when the embryo has inherited the *Cx3cr1*^{GFP} allele from the mother. Placenta macrophages are incompletely labeled, even when both mother and fetus express the *Cx3cr1*^{GFP} allele, reflecting the heterogenous expression of *Cx3cr1* (related to Fig. 2B). Data are represented as mean ± s.e.m of n = 4 embryos from two experiments (E12.5) and n = 4 embryos from two experiments (E14.5). (**D**) Representative gating strategy (left) from E10.5 placenta for quantification of microglia (CD45^{+} F4/80^{+} CD11b^{+}) labeling (right) in *Csf1r*^{MeriCreMer} *Rosa26*^{YFP} embryos with E8.5 OHT injection (related to Fig. 2C). Data are represented as mean ± s.e.m of n = 6 embryos from two experiments (E10.5) and n = 18 embryos from three experiments (E11.5). (**E**) Quantification of microglia and LSK (Lin^{neg} Sca-1^{+} Kit^{+}) labeling (top) in *Cdh5*^{CreERT2} *Rosa26*^{YFP} or *Rosa26*^{tdTomato} embryos with E7.5 OHT injection (related to Fig. 2E). Representative gating strategy for LSK (bottom) from E12.5 fetal liver. Data are represented as mean ± s.e.m of n = 6 embryos from two experiments (E12.5), n = 9 embryos from two experiments (E14.5) and n = 11 embryos from two experiments (E16.5). (**F**) Quantification of microglia and LSK labeling in *Cdh5*^{CreERT2} *Rosa26*^{YFP} embryos with E10.5 OHT injection (related to Fig. 2G). Data are represented as mean ± s.e.m of n = 8 embryos from two experiments (E12.5), n = 9 embryos from two experiments (E14.5) and n = 8 embryos from two experiments (E16.5). *P < 0.05, **P < 0.01, n.s., ***P < 0.001, n.s., not significant (Student's unpaired t-Test with Welch's correction).

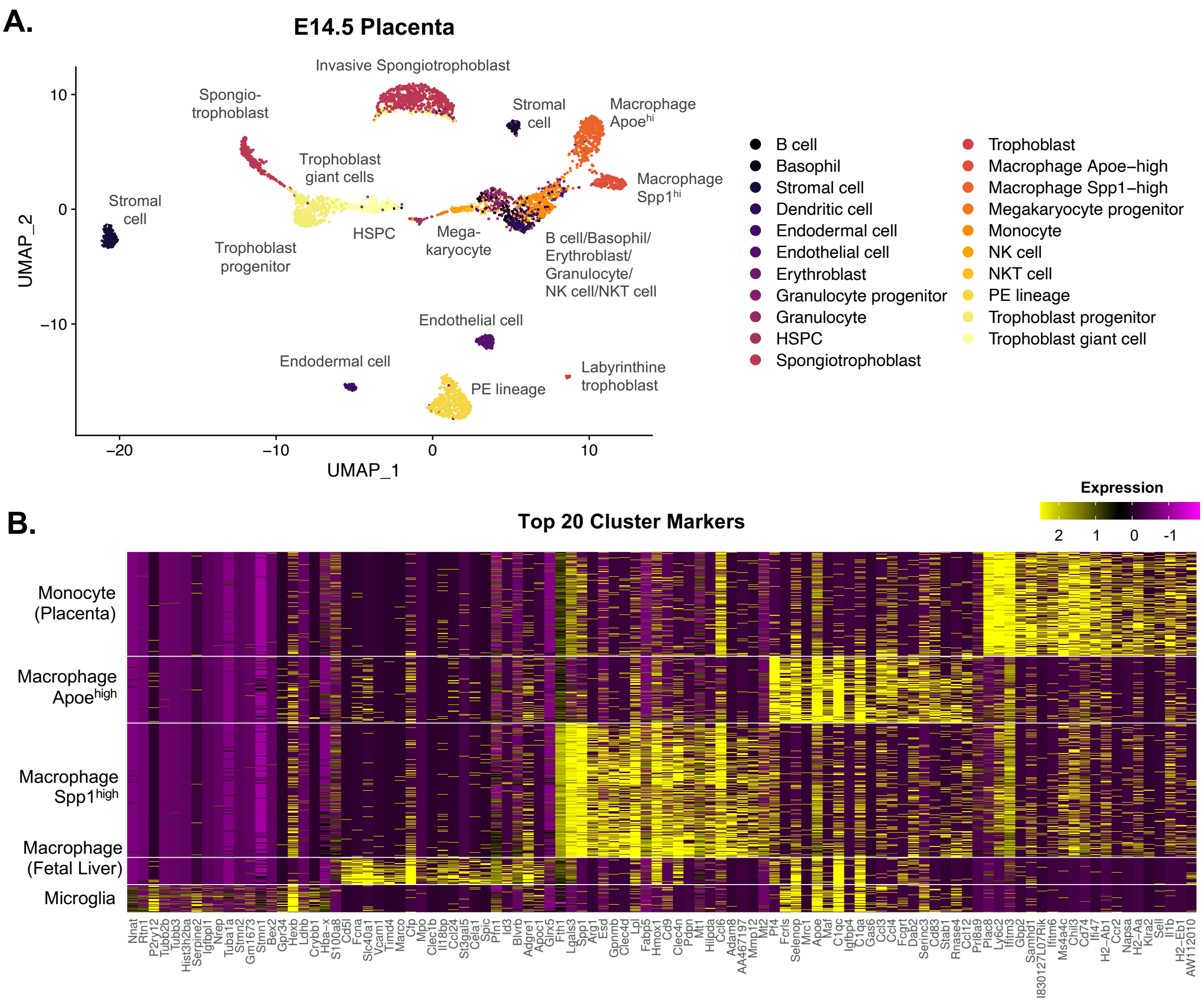

Fig. S3. Single-cell RNA sequencing of fetal and placenta macrophages. (**A**) Single-cell RNA-sequencing and clustering of 4,346 cells from the E14.5 mouse placenta color-coded according to placenta cell type (as assigned in Han *et al.*, 2018, related to Fig. 3A-B). Two distinct clusters of macrophages have been previously annotated as Apoe^{high} (192 cells) and Spp1^{high} (388 cells). (**B**) Top 20 cluster markers among monocytes and macrophages of the E14.5 mouse placenta, fetal liver and brain (related to Fig. C-E).

Development • Supplementary information

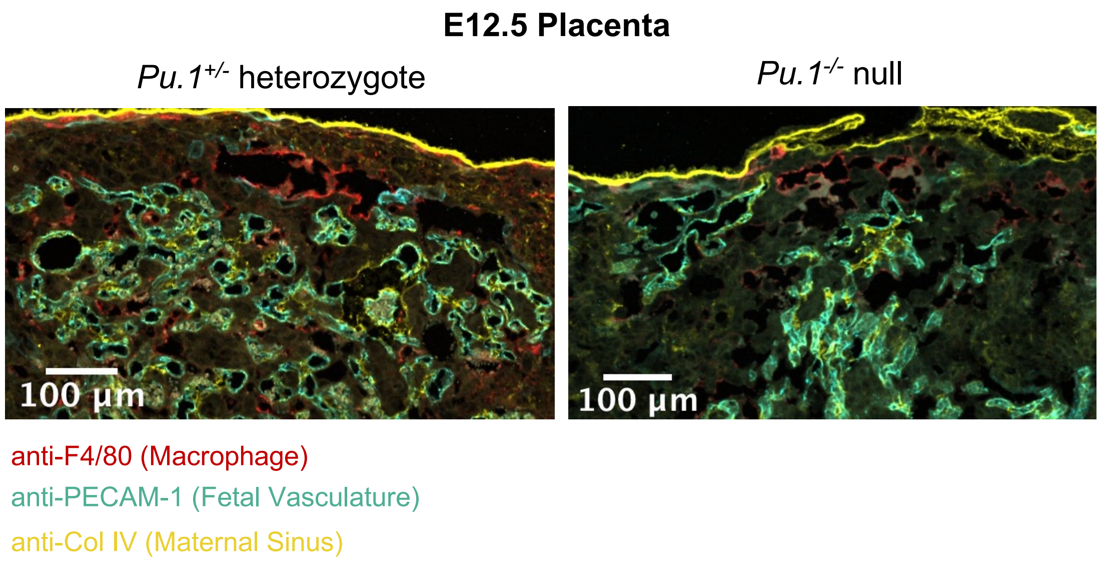

Fig. S4. Morphological defects in the E12.5 placenta of *Pu.1*$^{-/-}$ null mutants. Immunofluorescence on tissue sections of E12.5 placentas from *Pu.1*$^{+/-}$ heterozygous and *Pu.1*$^{-/-}$ homozygous null embryos. Macrophages of both fetal and maternal origin are stained by anti-F4/80. Fetal vasculature is stained by anti-CD31 while the lining of the maternal sinus is stained by anti-Col IV. Magnified fields were taken from the same region of the placental labyrinth (related to Fig. 4).

Development • Supplementary information

Table S1. Antibodies

Antibody	Fluorophore	Concentration	Clone	Supplier	Catalog Code
CD16/32	BUV737	1:100	2.4G2	BD Biosciences	565272
CD16/32	BV711	1:100	93	BioLegend	101337
CD45	BUV661	1:100	30-F11	BD Biosciences	612975
CD45	BUV395	1:100	30-F11	BD Biosciences	564279
CD45.2	APC-Cy7	1:100	104	Sony	1149120
CD41	BUV395	1:200	MWReg30	BD Biosciences	565980
F4/80	BV785	1:100	BM8	BioLegend	123141
F4/80	BV421	1:100	BM8	Sony	1215660
Ly-6G	BV711	1:100	1A8	BD Biosciences	563979
Ly-6C	BV605	1:100	HK1.4	Sony	1240180
Ly-6C	BV510	1:100	HK1.4	BioLegend	128033
Sca-1	BV510	1:200	D7	BioLegend	108129
Sca1	BV711	1:100	D7	BioLegend	108131
CD34	eF450	1:50	RAM34	eBioscience	15361710
Itgb7	PE-Cy7	1:100	FIB504	eBioscience	15598576
Ter119-biotin	-	1:50	TER-119	BioLegend	116204
CD19-biotin	-	1:200	1D3	BD Biosciences	553784
CD8-biotin	-	1:200	53-6.7	BioLegend	100704
CD4-biotin	-	1:100	H129.19	BD Biosciences	553649
CD3e-biotin	-	1:100	145-2C11	BD Biosciences	553060
NK1.1-biotin	-	1:100	PK136	BioLegend	108704
Ly6G-biotin	-	1:100	1A8	BioLegend	127604
CD115	PE-Dazzle594	1:100	AFS98	BioLegend	135527
Flt3	PE	1:100	A2F10	eBioscience	15258669
Kit	APC-Cy7	1:200	2B8	Sony	1129130
Kit	PE	1:200	2B8	BD Biosciences	553355
CD11b	AF700	1:200	M1/70	BD Biosciences	557960
CD11b	PE-Cy7	1:200	M1/70	BD Biosciences	552850
CD71	AF647	1:200	C2	BD Biosciences	563504
SAV	PE-Cy5	1:200	-	BioLegend	405205
SAV	BUV737	1:200	-	BD Biosciences	612775
Cx3cr1	PE	1:100	QA16A03	BioLegend	153705
Ki-67	AF647	1:50	B56	BD Biosciences	558615
Rat anti-F4/80	unconjugated	1:200	Cl:A3-1	BIO-RAD	MCA497G
Hamster anti-PECAM-1	unconjugated	1:250	2H8	Abcam	ab119341
Rabbit anti-Collagen IV	unconjugated	1:200	-	BIO-RAD	2150-1470
Goat anti-Hamster	Alexa Fluor 647	1:250	-	Interchim	127-605-160
Goat anti-Rat	Alexa Fluor 488	1:500	-	Abcam	ab150165
Goat anti-Rat	Alexa Fluor 555	1:500	-	Abcam	ab150165
Goat anti-Rabbit	Alexa Fluor 488	1:500	-	Invitrogen	A11034

Development: doi:10.1242/dev.200104: Supplementary information

Supplementary Materials and Methods

Gating strategies for flow cytometry

Monocytes and Macrophages: Lin^{neg} (Ter119^{neg} CD19^{neg} CD8^{neg} CD4^{neg} CD3e^{neg} NK1.1^{neg} Ly6G^{neg}) CD45^{+} CD16/32^{+}; Placenta macrophages: Lin^{neg} (Ter119^{neg} CD19^{neg} CD8^{neg} CD4^{neg} CD3e^{neg} NK1.1^{neg} Ly6G^{neg}) CD45^{+} CD16/32^{+} Ly6C^{neg} CD34^{+} F4/80^{+} Kit^{neg-lo}; Macrophage precursors: Lin^{neg} (Ter119^{neg} CD19^{neg} CD8^{neg} CD4^{neg} CD3e^{neg} NK1.1^{neg} Ly6G^{neg}) CD45^{+} CD16/32^{+} Ly6C^{neg} CD34^{+} Kit^{+} F4/80^{neg-lo} Cx3cr1-GFP^{lo}; Microglia: CD45^{+} F4/80^{+} CD11b^{+}; LSK: Lin^{neg} Sca-1^{+} Kit^{+}; Progenitor/precursor**:** Lin^{neg} (Ter119^{neg} CD19^{neg} CD8^{neg} CD4^{neg} CD3e^{neg} NK1.1^{neg} Ly6G^{neg}) CD45^{+} CD16/32^{+} Ly6C^{neg} CD34^{+} Kit^{+} F4/80^{neg-lo}.

Development • Supplementary information
